# Placental Histomorphology in a Case of Double Trisomy 48,XXX,+18

**DOI:** 10.1155/2018/2839765

**Published:** 2018-03-08

**Authors:** Sujal I. Shah, Lisa Dyer, Jerzy Stanek

**Affiliations:** ^1^Department of Pathology and Laboratory Medicine, University of Cincinnati Medical Center, Cincinnati, OH, USA; ^2^Division of Pathology and Laboratory Medicine, Cincinnati Children's Hospital Medical Center, Cincinnati, OH, USA; ^3^Division of Human Genetics, Cincinnati Children's Hospital Medical Center and University of Cincinnati College of Medicine, Cincinnati, OH, USA

## Abstract

**Background:**

Approximately 50% of early spontaneous abortions are found to have chromosomal abnormalities. In these cases, certain histopathologic abnormalities are suggestive of, although not diagnostic for, the presence of chromosomal abnormalities. However, placental histomorphology in cases of complex chromosomal abnormalities, including double trisomies, is virtually unknown.

**Case Report:**

We present the case of a 27-year-old G3P22002 female presenting at 19 weeks and 1 day of gestation by last menstrual period for scheduled prenatal visit. Ultrasound revealed a single fetus without heart tones and adequate amniotic fluid. Limited fetal measurements were consistent with estimated gestational age of 17 weeks. Labor was induced with misoprostol due to fetal demise. Autopsy revealed an immature female fetus with grade 1-2 maceration. The ears were low-set and posteriorly rotated. The fingers were short bilaterally, and the right foot showed absence of the second and third digits. Evaluation of the organs showed predominantly marked autolysis consistent with retained stillbirth. Placental examination revealed multiple findings, including focal pseudovillous papilliform trophoblastic proliferation of the undersurface of the chorionic plate and clustering of perpendicularly oriented sclerotic chorionic villi in the chorion laeve, which have not been previously reported in cases of chromosomal abnormalities. Karyotype of placental tissue revealed a 48,XXX,+18 karyotype and the same double trisomy of fetal thymic tissue by FISH.

**Conclusion:**

In addition to convoluted outlines of chorionic villi, villous trophoblastic pseudoinclusions, and clusters of villous cytotrophoblasts, the previously unreported focal pseudovillous papilliform trophoblastic proliferation of the undersurface of the chorionic plate and clustering of perpendicularly oriented sclerotic chorionic villi in the chorion laeve were observed in this double trisomy case. More cases have to be examined to show if the histology is specific for this double trisomy.

## 1. Background

First trimester evaluation for chromosomal abnormalities includes a combination of biochemical and ultrasound screening [1]. Chromosomal studies are frequently performed in early spontaneous abortions, without corresponding histological examination. Thus, there is not much literature correlating chromosomal abnormalities with specific histomorphologic findings [2]. In more advanced pregnancies, indications for a cytogenetic workup may include congenital anomalies and/or placental dysmorphology. As such, it is important to have an adequate understanding of what the histomorphology of a placenta looks like in situations of chromosomal anomalies. When malformations are absent or scant in this setting, placental morphology could potentially be an indication to perform further cytogenetic and molecular workup. This may be particularly true in cases of double trisomy X and 18, in which fetal features are dominated by those related to trisomy 18 [3] and those of trisomy X may be overlooked. For example, ear and reproductive malformations may be seen in 48,XXX,+18 [3]; in addition, polyhydramnios, fetal growth restriction, and enlargement of cisterna magna may also be seen [4].

Placental histomorphology in chromosomal anomalies shows many nonspecific findings, including small size of the placenta, villous edema, trophoblastic invaginations, and irregular villous contours [5]. In cases of chromosomal trisomy, single umbilical artery, deficient vascularization, and increased villous cellularity may be occasionally seen [2, 5, 6]. When found in spontaneous abortions, these abnormalities are suggestive of, although not diagnostic for, chromosomal abnormalities; however, little correlation has been made between findings and specific chromosomal anomalies. The findings are usually described in early spontaneous abortions, about 50% of which are found to have chromosomal abnormalities [7]. Little is known about placental morphology in late spontaneous abortions or more advanced pregnancies, and placental histomorphology in complex chromosomal abnormalities is virtually unknown.

Hereby we present a case of double trisomy 48,XXX,+18 diagnosed by karyotyping of placental tissue. Placental pathology of such cases has not been reported yet.

## 2. Case Report

A 27-year-old G3P22002 female presented at 19 weeks and 1 day of gestation by last menstrual period for scheduled prenatal visit. Ultrasound study was performed and revealed a single fetus and adequate amniotic fluid with limited fetal measurements consistent with estimated gestational age of 17 weeks, that is, an age 2 weeks less than the estimated gestational age. Diffuse subcutaneous edema was seen, with possible multiloculated nuchal cystic hygroma. Fetal heart tones could not be detected and a three-vessel umbilical cord could not be documented.

The maternal history included 2 previous uncomplicated term vaginal births of children with no anomalies. Age at menarche was 13 years. The mother is a nonsmoker and she was taking supplemental vitamins. She reported no alcohol, caffeine, or illicit drug intake. She is sexually active with a single partner. Most recent cervical pap smear was approximately 4 years ago, with normal results. Prior to this pregnancy, her periods were regular and heavy. Her IgG for rubella was positive approximately at 8 weeks' gestation. The patient denied any significant family history for congenital conditions.

Because of fetal demise, labor was induced with misoprostol and resulted in a spontaneous vaginal delivery of a stillborn female fetus followed by spontaneous delivery of the placenta. The fetus and placenta were transferred to our institution for autopsy and placental examination.

**Figure 1:**
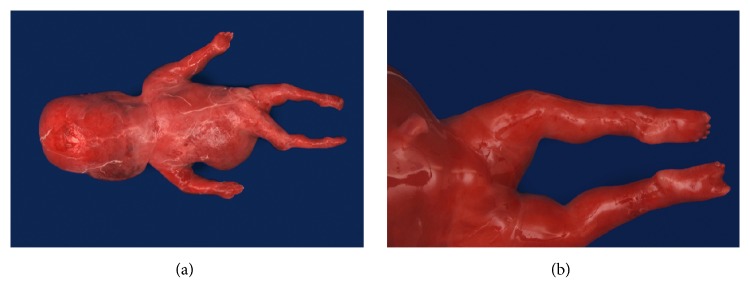
Hydrops, posterior nuchal fluid accumulation (a) and right foot, showing absence of second and third digits (b).

At autopsy, a slightly growth-restricted previable, immature female fetus with grade 1-2 maceration and moderate-to-marked hydrops was examined. The posterior nuchal fluid accumulation was prominent. The ears were low-set and posteriorly rotated. The fingers were short bilaterally (Figure 1(a)), and the right foot showed absence of the second and third digits (Figure 1(b)). The pulmonary artery was slightly narrow, with no other cardiovascular findings. Microscopic examination showed desquamated epidermis and nuchal edema. Evaluation of the organs showed predominantly marked autolysis consistent with retained stillbirth.

**Figure 2:**
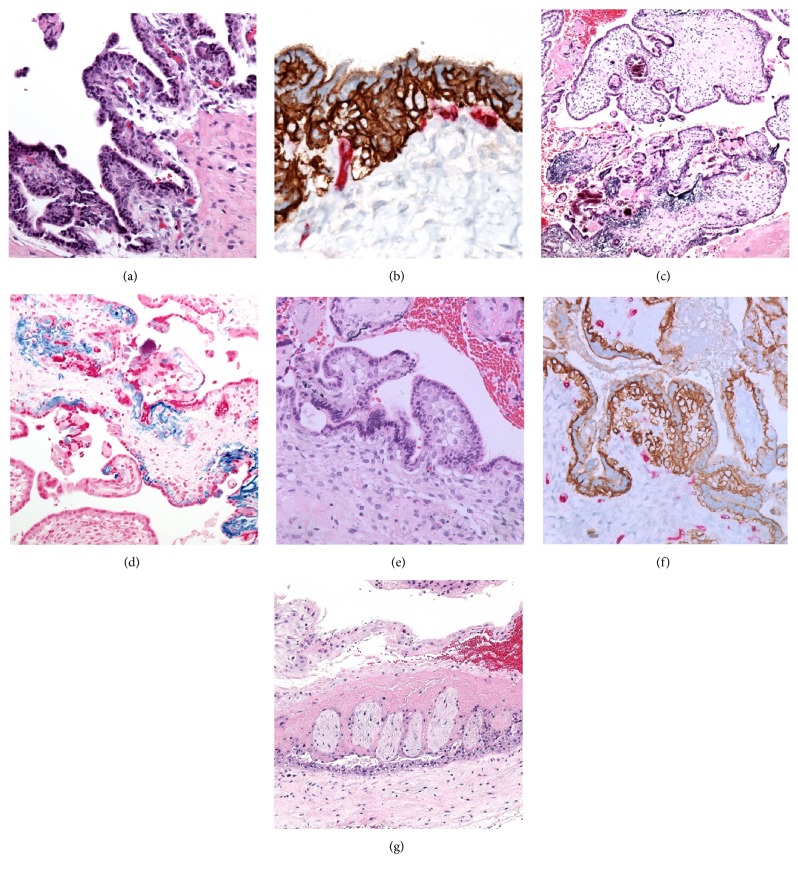
Histologic examination of the placenta. Pseudovillous trophoblastic proliferation present at the undersurface of the chorionic plate* [(a) magnification *×100*, H&E]*, highlighted by double E-cadherin (brown)/CD34 (red) immunostain* [(b) magnification *×400*]*. Large irregular chorionic villi, showing trophoblastic pseudoinclusions and focal mineralization* [(c) magnification *×100*, H&E]*. Iron stain highlights the ferrugination* [(d) magnification *×200*]*. Clusters of villous trophoblasts are seen* [(e) magnification *×400*, H&E]*, highlighted by dual E-cadherin (brown) and CD34 (red) immunostain* [(f) magnification *×400*]*. Perpendicularly oriented clusters of sclerotic chorionic villi are present in the chorion laeve* [(g) magnification *×100*, H&E]*.

Placental examination revealed membrane laminar necrosis, consistent with acute hypoxic injury. The undersurface of the chorionic plate showed focal pseudovillous papilliform cytotrophoblastic proliferation (Figure 2(a)). This was highlighted by double immunohistochemical stain for E-cadherin/CD34 (Figure 2(b)). The chorionic villi were large and cellular, with convoluted outlines and presence of both trophoblastic pseudoinclusions (Figure 2(c)), secondary to villous scalloping, and focal robust ferrugination of the basal lamina (Figure 2(c)), highlighted by iron stain (Figure 2(d)). Rare clusters of villous cytotrophoblasts were seen, with normal villous vascularity (Figure 2(e)), highlighted by E-cadherin/CD34 immunostain (Figure 2(f)). The number of cell islands was increased. The chorion laeve showed an uneven distribution of perpendicularly oriented and elongated clusters of sclerotic chorionic villi (Figure 2(g)).

**Figure 3:**
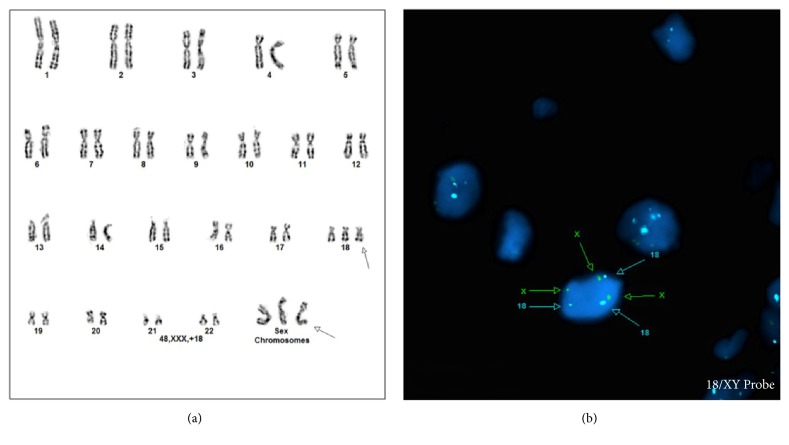
Karyotype of placental tissue reveals coexistent trisomy 18 and trisomy X (a). Fluorescence* in situ* hybridization performed on thymic tissue confirms presence of trisomy 18 and trisomy X (100/100 cells) (b).

G-banded karyotyping of placental tissue revealed a 48,XXX,+18 karyotype (Figure 3(a)). Due to maceration, karyotype of fetal tissue was not obtained. Fluorescence* in situ* hybridization testing for chromosomes X and 18 was performed on relatively well-preserved thymic tissue and highlighted double trisomy of these chromosomes (Figure 3(b)), which rules out an isolated placental mosaicism.

## 3. Discussion and Conclusions

Early spontaneous abortions (i.e., spontaneous abortions occurring in the first 12 weeks of gestation) occur in 20% of all pregnancies [8]. Of these, almost 50% have a chromosomal abnormality, and almost 50% of these abnormalities are chromosomal trisomies, most being single trisomies. Trisomy 18 (Edwards syndrome) has an estimated overall incidence of 1/2500 to 1/2600 pregnancies [3], while a 47,XXX trisomy has prevalence of 1/1000 in live births [9]. Consequently, double trisomy X/18 is significantly less common, and, as a result, only a handful of cases of double trisomy 48,XXX,+18 have been reported in the literature. The rate of all double trisomies in karyotyped spontaneous abortions is reported to range from 0.21% to 2.8% [10]. The incidence of double trisomy in later pregnancy is unknown.

**Figure 4:**
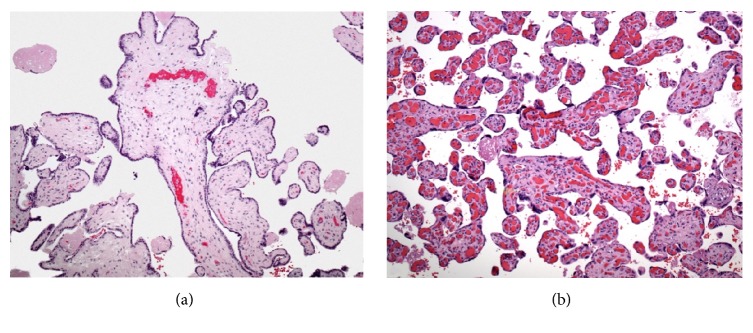
Trisomy 18: stillbirth at 34 weeks. Convoluted outlines of chorionic villi with trophoblastic pseudoinclusions* [(a) magnification *×100*, H&E]*. Trisomy X: focal chorangiosis, with no features of aneuploidy* [(b) magnification *×100*, H&E]*.

Typically, trisomy X is a maternal meiosis I error (63% of the time) and trisomy 18 is a maternal meiosis II error 59% of the time [11]. On the other hand, double trisomy involving chromosomes X and 18 has been reported to occur by nondisjunction in the second meiotic division [4, 11]. The affected individuals can survive postnatally [12]; the life span is shortened [13], although not extremely shortened in comparison to cases of trisomy 18 [14]. Most commonly, the condition is diagnosed by karyotyping of spontaneous abortion specimens; but placental histology in such cases has not been reported [10, 15]. Placental histology was studied in trisomy 18, which featured cystic and dilated chorionic villi with hydropic change [16]. Cysts may be large and identified grossly; there may be an increase in density of syncytial knots or in cytotrophoblast clustering within the villous stroma [2, 16], as well as placental dysmaturity [17]. Decreased villous vascularity and basophilic stippling in the basement membrane has also been observed [18]. Figures 4(a) and 4(b) present the villous histology of two unrelated cases of trisomy X and trisomy 18. There are no reports on double trisomy X and 18 with results of placental histology.

We regard the above-mentioned “basophilic stippling” of villous basement lamina a nonspecific finding which is usually positive for iron; hence we call it ferrugination [19]. It is not uncommonly seen in hydropic placentas, in retained stillbirth [20], and in fetal thrombotic vasculopathy/villous hypoperfusion, albeit, in this setting, it is not diffusely seen but in a lobular distribution [19]. The latter may be relevant, as placentas of trisomy 18 may show features of fetal thrombotic vasculopathy/decreased fetal blood perfusion including occlusion, recanalization, and calcification of vascular walls [17]. As isolated trisomy X shows no distinct malformations at birth except for minor abnormalities such as epicanthal fold and clinodactyly, trisomy X may be totally asymptomatic at birth and likely diagnosed later in life [9].

In addition to convoluted outlines of chorionic villi, villous trophoblastic pseudoinclusions, and clusters of villous cytotrophoblasts, the previously unreported focal pseudovillous papilliform trophoblastic proliferation of the undersurface of the chorionic plate and clustering of perpendicularly oriented sclerotic chorionic villi in the chorion laeve were observed in this double trisomy case. Whether it is characteristic of the underlying double trisomy is unknown and it must be confirmed in more double trisomy cases.

In summary, fetal malformations in our case were scant, exhibiting a potential discrepancy between placental and fetal findings. The placental examination revealing previously unreported placental histomorphologic features in addition to the known background features of aneuploidy may be significant. However, this finding must be independently confirmed, particularly as there is a substantial interobserver variability in evaluating placental histomorphology in chromosomal abnormalities [3].
